# Activatable cell-penetrating peptides: 15 years of research

**DOI:** 10.1039/d0cb00114g

**Published:** 2020-08-26

**Authors:** Heleen de Jong, Kimberly M. Bonger, Dennis W. P. M. Löwik

**Affiliations:** Department of Synthetic Organic Chemistry, Institute for Molecules and Materials, Radboud University Nijmegen The Netherlands k.bonger@science.ru.nl d.lowik@science.ru.nl

## Abstract

An important hurdle for the intracellular delivery of large cargo is the cellular membrane, which protects the cell from exogenous substances. Cell-penetrating peptides (CPPs) can cross this barrier but their use as drug delivery vehicles is hampered by their lack of cell type specificity. Over the past years, several approaches have been explored to control the activity of CPPs that can be primed for cellular uptake. Since the first report on such activatable CPPs (ACPPs) in 2004, various methods of activation have been developed. Here, we provide an overview of the different ACPPs strategies known to date and summarize the benefits, drawbacks, and future directions.

## Abstract 2

Since the first report on activatable CPPs (ACPPs) in 2004, various methods of activation have been developed. Here, we provide an overview of the different ACPP strategies known to date and summarize the benefits, drawbacks, and future directions.

## Graphical abstract

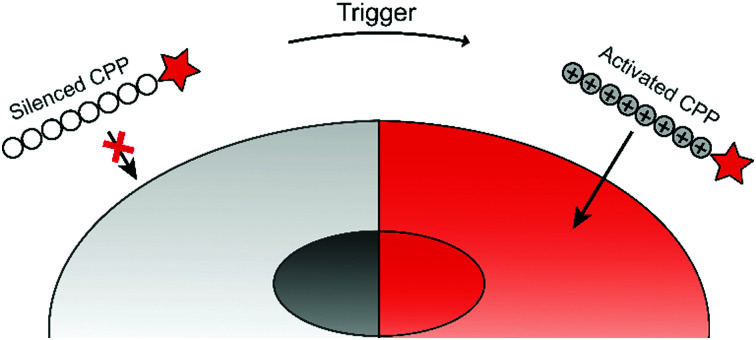

## Introduction

Cells have a complex and mostly impermeable cell membrane to ensure stable intracellular conditions and to protect the cell from harmful exogenous substances. This lipid bilayer membrane allows the diffusion of small molecules but excludes the entry of larger molecular entities. As a consequence, hydrophilic small molecules and protein- or nucleic acid-based therapeutics are also excluded and effectively transferring these over the cell membrane has been a challenge for many years.^1^

A promising method for the intracellular delivery of membrane-impermeable therapeutics emerged with the discovery that certain peptides could transfer cargo across the cell membrane. In 1988, two separate research groups reported that human influenza virus (HIV) Tat trans-activator proteins enabled cellular uptake along with the transport of cargo.^2,3^ Green and Loewenstein also identified the primary sequence of the uptake region of Tat (RKKRRQRRR).^2^ Since these first reports, many other so-called cell penetrating peptides (CPPs) have been identified including Antennapedia homeoprotein derived Penetratin,^4^ Arf(1–22) and M918 derived from p14Arf,^5,6^ and Xentry derived from the hepatitis B virus (Table 1, entries 1–5).^7^ In addition, several synthetic CPPs have been designed containing polybasic or hydrophobic residues, including polyarginine,^8^ Pip,^9^ CADY,^10^ and others (Table 1, entries 6–8).

**Table 1:** Overview of several common natural derived and synthetic cell-penetrating peptides (CPPs)

	Name	Sequence	Source	Ref.
Natural occurring CPPs				
1	Tat	RKKRRQRRR	HIV	2 and 3
2	Penetratin	RQIKIWFQNRRMKWKK	Antennapedia	4
3	Arf(1–22)	MVRRFLVTLRIRRACGPPRVRV	p14Arf	5
4	M918	MVTVLFRRLRIRRACGPPRVRV	p14Arf	6
5	Xentry	LCLRPVG	Hepatitis B virus	7

Synthetic CPPs				
6	PolyArg	R_*n*_ (*n* > 6)	NA	8
7	Pip	(RXR)_3_IKILFQNRRMKWKK	NA	9
8	CADY	GLWRALWRLLRSLWRLLWKA	NA	10

**Fig. 1:**
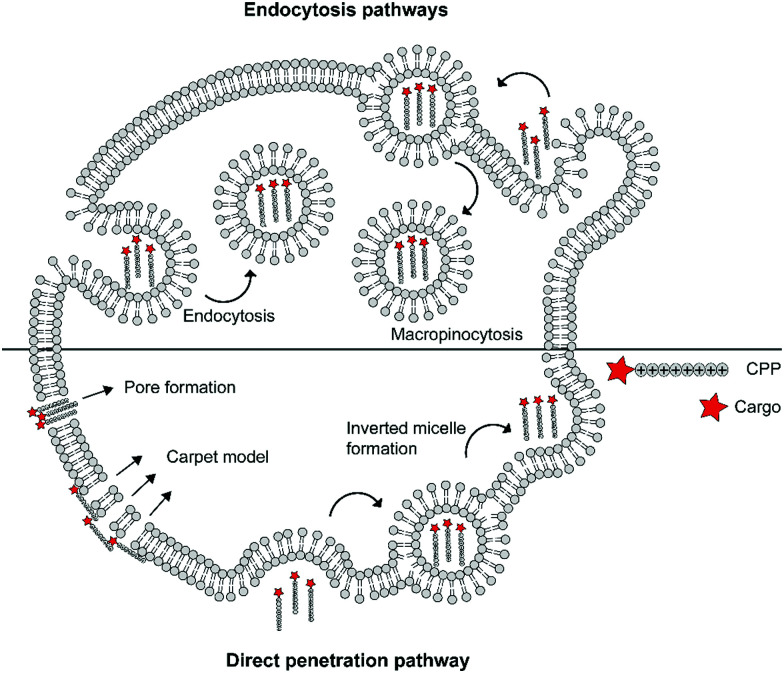
Schematic summary of various mechanisms of cellular uptake of cationic cell-penetrating peptides (CPPs) via endocytic pathways or direct penetration.

The cellular uptake mechanisms of CPPs have been extensively studied, but are still poorly understood as different CPPs seem to enter cells through different pathways.^11,12^ Mechanisms of uptake can be classified in two categories: energy independent direct penetration and energy dependent endocytosis (Fig. 1). Direct penetration occurs in cases when a high concentration of peptide is available and involves a tight interaction between the CPPs and cell membrane. Direct cell penetration mechanisms include (1) pore formation, where the CPPs insert themselves in the membrane; (2) the carpet model, where CPPs position on the membrane as a carpet, thereby increasing membrane fluidity and passage of the CPPs; and (3) inverted micelle formation, where the phospholipid bilayer encapsulates the peptide by formation of inverted micelles. However, CPPs, especially those that carry cargo, internalize mainly through endocytic pathways.^13^ These include micropinocytosis, clathrin- or caveolin-mediated endocytosis, and clathrin/caveolin independent endocytosis.^14^ The path taken depends on the size and physicochemical properties of the cargo as well as the nature of the CPP and the target cell.^15^ For more detailed information on the uptake mechanisms of CPPs and the contributing factors we refer to some recent reviews on this topic.^12,16^

Even though CPPs are potentially promising drug delivery vehicles, several issues hamper their use in practice. These include (1) their lack of cell type specificity resulting in uncontrolled uptake and potential adverse effects,^17^ (2) their toxicity at high concentrations, which is associated with membrane perturbation^18^ and (3) their fast blood clearance as was shown by a study where only 1% of the injected dose of ten different CPPs remained at the target site after 4 h in tumour bearing mice.^17^

**Fig. 2:**
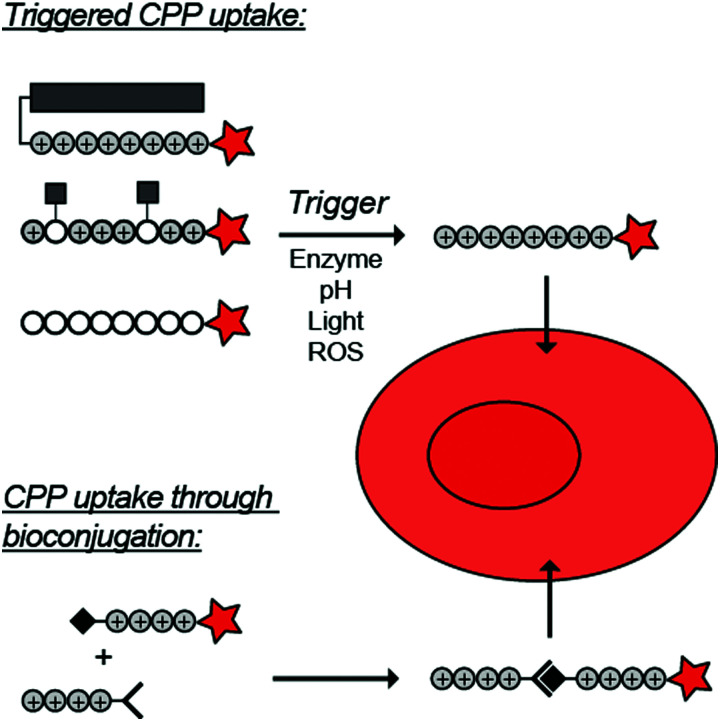
Dormant cell-penetrating peptides (CPPs) cannot enter cells. Upon triggering they are activated and can be taken up by cells.

To overcome these challenges, much research is focussed over the last years to control CPPs and to activate them only at the target site by use of an external trigger (Fig. 2). The Tsien group first described such so-called Activatable CPPs (ACPPs) in 2004 where they fused a polycationic CPP to an inhibiting polyanionic domain *via* a protease cleavable linker.^19^ Since then, numerous advances in the development of ACPPs and their triggers have been reported. In this review, we provide an overview of the strategies in the design of ACPPs since their discovery more than 15 years ago.

## CPP activation through removal of an inhibitory domain

Cationic CPPs interact with the negatively charged cell membrane via electrostatic attraction. Hence, masking the positive charge using an inhibitory domains restrains cellular uptake.^15,19^ Inhibitory domains that have been explored include poly(ethylene) glycol (PEG) blocks that sterically mask the CPP, or polyanionic domains that lower the isoelectric point of a cationic peptide. The inhibitory domains are conjugated to the CPP *via* a trigger responsive linker to control cellular uptake. Below and in Table 2, we summarize the various reported approaches in more detail.

**Table 2:** Activatable cell-penetrating peptides (ACPPs) based on linkage of an inhibitory domain. Amino acids are indicated via the single letter code, in which d-amino acids are noted in lower case. Cy5 = cyanine5 red dye; PpIX = protoporphyrin IX; Dox = doxorubicin; PLK-1 = polo-like kinase 1; PSA = protease specific antigen; NE = neutrophil elastase; FITC = fluorescein; NA = not available; CPT = camptothecin; EGFR = epidermal growth factor; QDs = quantum dots

	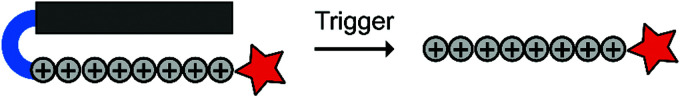					
	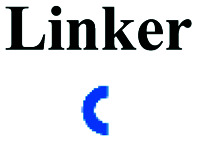	CPP	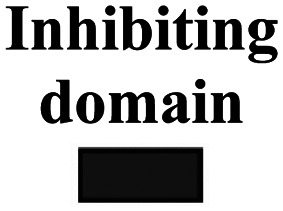	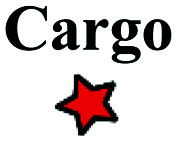	Trigger	Ref.
Enzyme sensitive linkers						
1	PLGLAG	r_9_	e_6,8_	Cy5	MMP-2 and -9	19 and 21
		R_9_	E_8_	PpIX		23
		R_9_	E_9_	Dox		24
		Poly(M-*block*-K)	PEG	Dox		25
2	PVGLIG	R_9_	(EGG)_3_	CsA-LMNC	MMP-9	26
3	PGFK	Tat	E_6_	Dox	Cathepsin B	28
4	HSSKYQ	R_8_	(DGG)_4_	PLK-1 siRNA	PSA	29
5	RLQLK(Ac)L	r_9_	e_9_	Cy5	NE	32
6	DPRSFL	r_9_	e_8_	Cy5 Rhodamine	Thrombin	33

pH sensitive linkers						
7	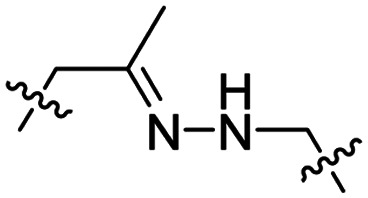	R_8_	(ehG)_4_	PLK-1 siRNA	pH < 6.8	37

ROS sensitive linkers						
8	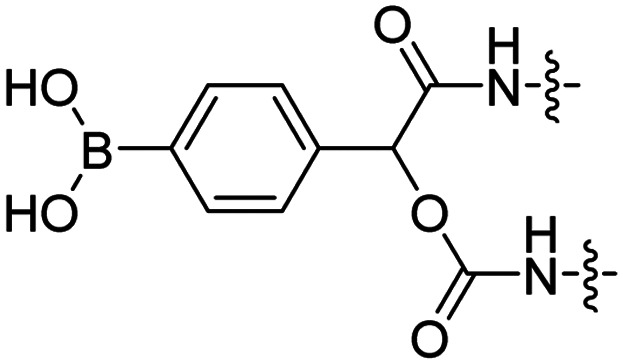	R_9_	E_9_	FITC Cy5	H_2_O_2_	43

Light sensitive linkers						
9	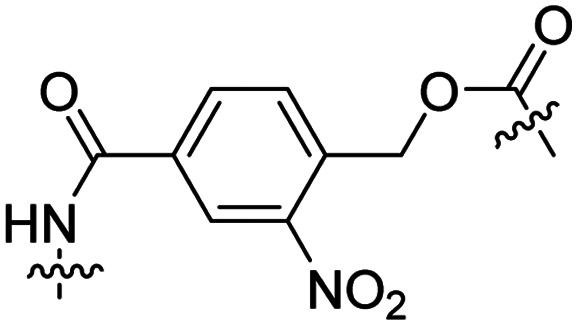	Tat	PEG	Liposomes loaded with Atto655	*λ* = 254 nm, 2 min	47
10	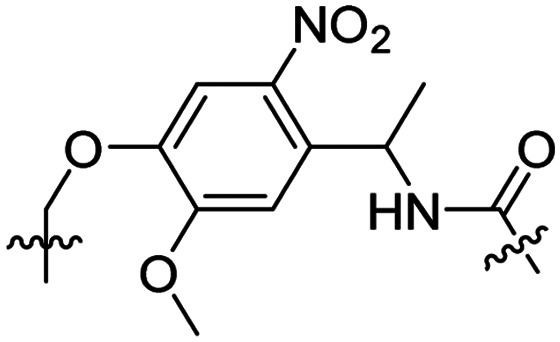	R_7_	E_7_	FITC CPT	*λ* = 365 nm, 10 min	48
11	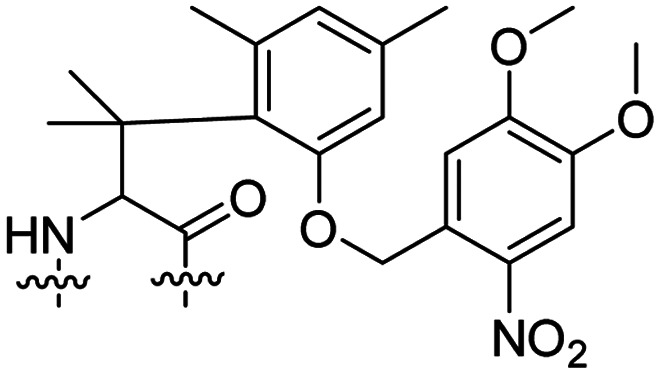	Penetratin	E_4_R_4_	EGFR siRNA	*λ* = 740 nm, 30 min and pH < 6.4	53
12	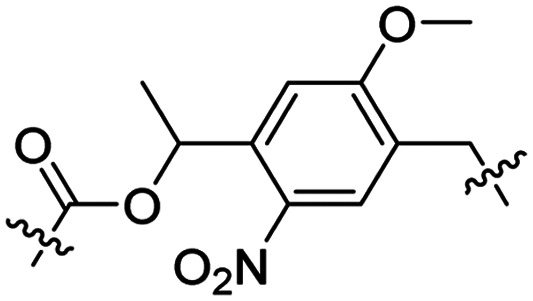	R_7_	E_7_	QDs	Two-photon, *λ* = 740 nm, 3 h	48

### Enzyme triggered removal

Compared to healthy tissue, diseased tissue may abundantly expresses particular proteolytic enzymes such as cathepsins, urokinases, caspases and matrix metalloproteases (MMPs).^20^ The earliest example on ACPPs by the Tsien group exploited an MMP-sensitive PLGLAG linker to connect a polycationic CPP to a polyanionic inhibitory domain.^19^ Enzymatic cleavage of the linker resulted in dissociation of the inhibitory domain and specific CPP uptake in MMP rich fibrosarcoma cells with an 18-fold difference between activated and non-activated structures (Table 2, entry 1).^19^ In a follow-up study, similar structures exhibited reduced blood clearance, improved distribution to the target site, and reduced toxicity compared to CPP controls.^21^ The Tsien group also showed that MMP-sensitive ACPPs are taken up in a variety of tumors *in vivo*.^22^

Using a similar ACPP construct with the MMP-sensitive linker, the Zhang group delivered a protoporphyrin photosensitizer to fibrosarcoma cells *in vivo*.^23^ Imaging experiments demonstrated efficient localization to tumours and a decrease in tumour size upon a 12-day treatment of injections once every three days. Alternatively, the group of Cao delivered doxorubicin (DOX) to human breast adeno-carcinoma cells, but not to MMP deficient human mammary endothelial cells, by coating DOX-loaded nanoparticles with MMP-triggered ACPPs.^24^ The Kim group combined MMP-responsiveness with reactive oxygen species (ROS) triggers to additionally advance the control of drug delivery.^25^ In this case, micelles were coated with ACPPs based on (poly(l-methionine-*block*-l-lysine)–PLGLAG–PEG) and loaded with DOX or IR-760 dye. MMP-assisted PLGLAG cleavage activated the polylysine-based ACPP to facilitate cellular uptake after which ROS oxidized the methionine thioether moieties to hydrophilic sulfoxide groups thereby destabilizing the micelle structures. High accumulation in tumours and a long retention time were observed, as well as efficient ROS triggered cargo release.^25^

Chen and coworkers coated a cyclosporine A loaded nanocarrier with an ACPP connected via an alternative MMP sensitive linker (PVGLIG) and studied the localization in controlled cortical impact injury mice (Table 2, entry 2).^26^ The structures crossed the blood brain barrier and internalized into primary astrocytes and neurons at the lesion site. Cyclosporine A inhibits the opening of the mitochondrial permeability transition pore thereby preventing apoptosis. Less cell death was observed around the lesion site in mice treated with the cyclosporine A-loaded and ACPP-coated nanocarriers compared to the cyclosporine A treated controls.

Besides MMPs, a cathepsin B cleavable sequence, PGFK, has been used to connect a polyanionic inhibitor to a Tat derived CPP that was conjugated to mesoporus silica quantum dot nanocarriers loaded with DOX (Table 2, entry 3).^27,28^ In the presence of endogenous cathepsin B levels, DOX was released and transported selectively to the nuclei of human adenocarcinoma cells while uptake was drastically decreased in the absence of cathepsin B.

Xiang and coworkers used the prostate-specific antigen (PSA), which is overexpressed in prostate cancer, as trigger to specifically deliver siRNA against the polo-like kinase 1 (PLK-1) transcript. PLK-1 induces apoptosis in cancer cells when depleted.^29,30^ The authors coated siRNA loaded liposomes with ACPPs containing a PSA-responsive HSSKYQ linker between a polycationic CPP and a polyanionic peptide inhibitor (Table 2, entry 4). Flow cytometry and imaging studies indicated increased uptake in PSA rich human prostate cancer 22Rv1 cells compared to PSA deficient PC-3 cells. Moreover, PLK-1 expression was decreased in the prostate cancer cell line treated with the liposomes, but not in the untreated control. In these experiments, increased apoptosis accompanied PLK-1 downregulation. *In vivo* studies with 22Rv1 xenograft tumours showed increased tumour accumulation for ACPP coated liposomes, compared to polycationic CPP coated liposomes.

Neutrophil elastase is highly abundant in several cancers, such as human breast and lung cancer.^31^ The Tsien group included a neutrophil elastase sensitive RLQLK(Ac)L sequence in an ACPP design to visualize tumours with a Cy5 dye (Table 2, entry 5).^32^ The acetylated lysine improved specificity for the neutrophil elastase over other endogenously expressed elastases. Injection of ACPPs in nude mice with human breast cancer xenografts visualized the tumour 6 h after injection, while the structure was not observed in mice treated with control structures where the inhibiting domain was linked via a non-cleavable d-amino acid based sequence (rlqlkl).

The Tsien group further extended the ACPP concept using a thrombin-sensitive DPRSFL linker between a fluorescently labelled cationic CPP and an anionic inhibitory domain for imaging purposes (Table 2, entry 6).^33^ Thrombin is active in blood coagulation and abundant in atherosclerotic plaques, which narrow arteries.^34,35^ Gel electrophoresis showed that purified thrombin could cleave the ACPP *in vitro*. The ACPP was injected in mice with induced atherosclerosis and 6 h after injection, a positive fluorescence correlation was observed with plaque burden for the ACPP but not for non-cleavable controls. Fluorescence distribution was also increased in slices of human atheromas that were treated with the ACPP *ex vivo*, but not for those treated with the non-cleavable control.

### pH triggered removal

Due to the high energy demand of tumours, their ATP is predominantly generated by aerobic glycolysis producing lactic acid that gives rise to an acidic extracellular environment.^36^ This altered metabolism of carcinogenic tissue provides a promising strategy for CPP activation.

Hydrazones rapidly hydrolyse to a ketone and a hydrazine under acidic conditions and were included in the design of acid-sensitive ACPPs. Here, Xiang and coworkers coated PLK-1 siRNA loaded liposomes with hydrazone based ACPPs, where the hydrazone linked a polyanionic inhibiting domain to the polycationic CPP (Table 2, entry 7).^37^ Lowering the pH from 7.4 to 6.8 resulted in loss of the inhibitory domain, a decrease in PLK-1 mRNA levels, reduced PLK-1 protein expression and a significant increase in apoptosis, suggesting successful release of the siRNA.

### ROS triggered removal

Reactive oxygen species (ROS), such as H_2_O_2_, are upregulated in many diseases including diabetes,^38^ cardiovascular diseases,^39^ neurodegenerative disorders,^40^ and cancer.^41,42^ The Tsien group designed a ROS-sensitive ACPP by introducing a 4-boronic mandelic acid moiety between a cationic CPP and anionic inhibitory domain (Table 2, entry 8).^43^ The ACPP was further equipped with fluorescein (FITC) on the CPP and a cyanine5 red dye (Cy5) on the inhibitory domain. Using this construct, ROS levels could be measured via a change in fluorescence resonance energy transfer (FRET) signal as ROS exposure results in formation of a phenolate which undergoes 1,6-elimination and releases CO_2_ to liberate the Cy5-modified inhibitory domain. A 2.5-fold increase in FITC/Cy5 emission was seen in HL-60 cells where endogenous H_2_O_2_ expression was induced compared to non-induced controls. *In vivo*, a 2-fold increase in emission ratio was observed upon ACPP administration in mice with induced lung inflammation versus healthy controls.

### Light-triggered removal

Besides exploiting endogenous triggers present in cellular microenvironments, external triggers such as light have also been explored to activate CPPs on demand at a specific location. Several UV-sensitive linkers are based on a labile *o*-nitrobenzyl group that undergoes a radical rearrangement upon irradiation, resulting in cleavage of an amide, ester or carbamate moiety.^44–46^ In case of the latter, rearrangement leads to an additional favourable loss of CO_2_ before exposing the leaving group. The Löwik group used such an *o*-nitrobenzyl carbamate as a photocleavable linker (PCL) to bury lipidated, Tat-derived CPPs into PEG-coated liposome membranes (Table 2, entry 9).^47^ UV irradiation for two minutes resulted in linker cleavage allowing Tat to escape the steric crowd of the PEG coat and exerting its cell-penetrating properties. Compared to control liposomes, a 15-fold increased particle internalization in HeLa cells was observed as evidenced by flow cytometry analysis and confocal microscopy. The Stevens group conjugated a polyanionic domain to a CPP *via* an *o*-nitrobenzyl-based PCL and connected this structure to camptothecin loaded poly(lactic-*co*-glycolic acid) (PLGA) nanoparticles (Table 2, entry 10).^48^ Using these particles, enhanced cell death of human breast adenocarcinoma and HeLa cells was observed when they were irradiated with UV light for 10 minutes (*λ* = 365 nm), while no cell death was seen in the absence of light, demonstrating successful conditional CPP uptake and drug release.

The use of high energy UV light to activate ACPPs is less desirable for use *in vivo* as it has low penetration depth and damages tissue.^49,50^ Light triggered ACPPs based on low energy near-infrared (NIR) light have also been explored.^51^ NIR can cleave NIR-sensitive PCLs at the target site or UV-sensitive PCLs using two-photon excitation. In two-photon excitation, short light pulses of low-energy photons reach the target site quasi-simultaneously to generate enough energy to give rise to bond cleavage.^52^ The Mei group obtained a PCL by inclusion of a trimethyl lock, which increases reactivity through a favourable ring closing reaction.^53,54^ This PCL was used to connect a penetratin derived CPPs with a pH-sensitive inhibitory domain and these structures were coated onto siRNA-loaded nanocarriers (Table 2, entry 11). Cellular imaging studies in human breast adenocarcinoma cells indicated that both two-photon irradiation (*λ* = 740 nm) and a pH change were required for internalization, which led to reduced levels of the corresponding mRNA. Furthermore, ACCPs based on an ester bound *o*-nitrobenzyl PCL could be activated by two photon light (*λ* = 740 nm, 16 mW), which resulted in successful uptake in HeLa cells as evidenced by cellular imaging (Table 2, entry 12).^48^

## CPP activation through removal of side chain modifications

Besides using inhibitory domains, CPPs can also be inactivated by modifying the residue side chains. These modifications can again be removed by enzymes, altering the pH, or light triggers. The reported strategies using such direct modifications are summarized below and outlined in Table 3.

**Table 3:** Overview of activatable cell-penetrating peptides (ACPPs) based on side chain modifications. Amino acids are indicated via the single letter code, in which d-amino acids are noted in lower case. FITC = fluorescein; Luc PNA = luciferase peptide nucleic acid; Dox = doxorubicin; pen = penetratin; VB = vinorelbine bitartrate

	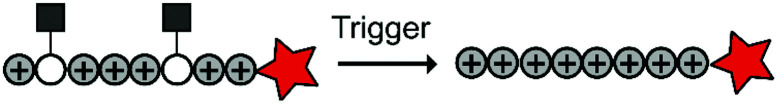					
	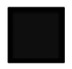	Number of R groups	CPP	Cargo	Trigger	Ref.
Enzyme sensitive side chain modifications						
1	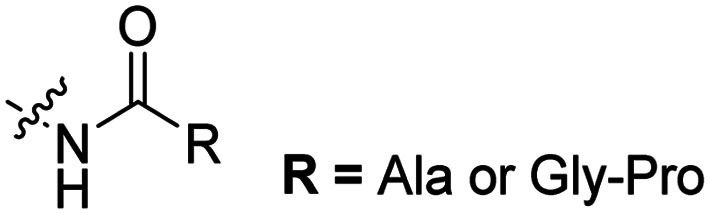	1 or 2	Tat	FITC	Aminopeptidase N dipeptidyl peptidase IV	55
2	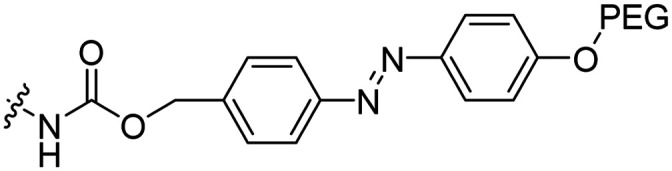	2	M918	Luc PNA	Bacterial azoreductase	57

pH sensitive side chain modifications						
3	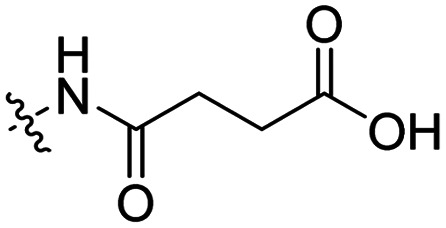	3	Tat	Dox	pH 5.0	58
4	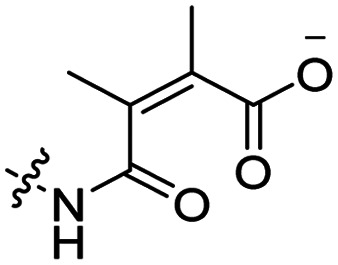	6	CR_8_G_3_PK_6_	Dox	pH 6.8	59

Light sensitive side chain modifications						
5	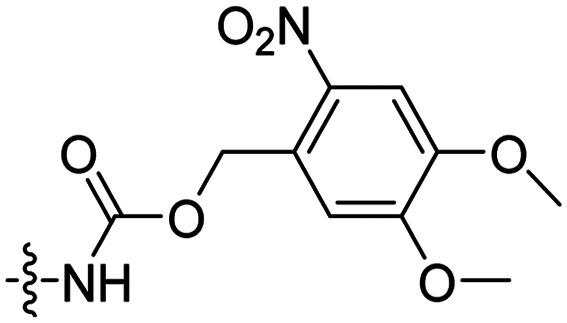	3	Pen	(klaklak)_2_	*λ* = 365 nm, 6 W, 10 min	60
6	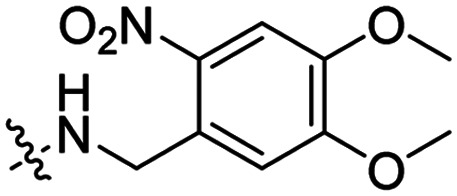	3	Pen	VB	*λ* = 740 nm, 30 min, 3.48 × 10^12^ photons s^−1^	61

### Enzyme triggered release

The Löwik group modified the lysine residues of a Tat peptide with an alanine or glycyl-proline motif to allow enzymatic CPP activation by aminopeptidase N or dipeptidyl peptidase IV, respectively (Table 3, entry 1).^55^ Single and double side chain modifications inhibited uptake, where a single alanine modification resulted in the largest decrease in cell uptake. Interestingly, the modifications did not remove the charge of the side chain completely, indicating that p*K*_a_, steric factors or spatial orientation of the charges may contribute to CPP uptake behaviour. ACPPs that were exposed to one of the peptidases displayed uptake in HEK cells, while unexposed ACPPs did not.

Leroux *et al.* explored an activation strategy using bacterial azoreductases, which are found in human colon mucosa and cleave azobenzene structures.^56^ Their synthetic CPPs were inactivated by conjugating PEG fragments to the side chains via an azobenzene moiety (Table 3, entry 2).^57^ As a proof of principle, a silencing peptide nucleic acid (PNA) specific for the firefly luciferase gene was conjugated to the C-terminus of the ACPP to monitor cell entry in luciferase-expressing colon adenocarcinoma cells. Incubation of the cells for two days resulted in azobenzene cleavage and 1,6-elimination followed by cell uptake of the CPP–PNA conjugate, as evidenced by a 40% decrease in luciferase activity compared to control cells.

### pH triggered release

The Murdoch group reported on the control of CPP activity by using acid sensitive side chain modifications. Here, they conjugated succinyl moieties to the glutamine and both lysine residues of Tat and used these to coat PEG-based micelles loaded with Nile Red dye or DOX (Table 3, entry 3).^58^ These ACPP-coated micelles were not taken up at physiological pH, while an 8 hour incubation at pH 5.0 resulted in cellular uptake comparable to that of micelles coated with unmodified CPPs. The ACPP-coated micelles displayed reduced blood clearance and increased localization to tumour cells *in vivo*, compared to unmodified CPP-coated controls. Furthermore, the tumour size in these mice was reduced upon treatment with DOX loaded structures.

Using a similar approach, Cheng *et al.* conjugated 2,3-dimethyl-maleic acid (DMA) to the lysine residues of a cationic CPP peptide (Table 3, entry 4).^59^ The DMA-linked lysine residues underwent electrostatic interactions with the arginine residues in the chain, thereby inactivating the ACPP. The labile amides hydrolysed at pH 6.8 and the DOX-linked ACPPs were efficiently taken up at this pH. Furthermore, they demonstrated that nonspecific cellular toxicity decreased, while repression of tumour growth resembled that of free DOX.

### Light triggered release

In a light sensitive ACPP design, the group of David modified the lysine side-chains of Penetratin with an *o*-nitrobenzyl moiety and loaded these ACPPs onto FITC-labelled *N*-(2-hydroxypropyl)-methacrylamide (HPMA) based polymers along with a proapoptotic peptide known as klak (Table 3, entry 5).^60^ Irradiation with UV light removed the photocage thereby allowing the CPP modified polymer to enter human prostate cancer cells, while polymers that were not illuminated did not show much cellular uptake.

Similarly, the Mei group conjugated *o*-nitrobenzyl photocages to the lysine residues of a Penetratin derived CPP and coated these onto liposomes loaded with anticancer agent Vinorelbine (VB, Table 3, entry 6).^61^ Two-photon NIR light activation (*λ* = 740 nm, 30 min) of the ACPP-coated liposomes resulted in uptake into human fibrosarcoma cells accompanied by a decreased viability and indicating that cargo could be delivered effectively.

## CPP activation through an induced conformational change

Changes in peptide conformation may affect the uptake properties of CPPs and have been explored to control CPP activity.^62^ Below we describe approaches where the conformation, and thus the activity of CPPs, can be tuned in response to triggers such as pH or light, without altering the primary peptide structure.

### pH triggered conformational changes

Histidines can serve as a pH trigger as their imidazole side chains have a p*K*_a_ around 6 and they are mostly unprotonated at physiological pH, while they can become protonated in an acidic environment. Lee and coworkers exploited this property and coated micelles with Tat-derived CPPs that were conjugated to long histidine repeats and PEG domains.^63^ At physiological pH, the neutral histidine domains interacted with the micelles to bury Tat between the PEG domains. Upon acidification, the histidine residues became protonated, lost their hydrophobic interactions and exposed Tat (Table 4, entry 1). Acidification of the extracellular fluid from 7.4 to 7.0 or 6.8 was accompanied by a 30- or 70-fold increase in uptake in human breast cancer cells, respectively.

**Table 4:** Overview of activatable cell-penetrating peptides (ACPPs) triggered through conformational changes. Amino acids are indicated *via* the single letter code. PEG = poly(ethylene) glycol; CPT = camptothecin; PTX = paclitaxel; probe = IR-probe; SC= side chain; LK = leucine and lysine rich CPP; *trans*-Ab = *trans*-azobenzene; Tamra = Tamra red dye; RhoB = rhodamineB dye; y^Ahx^ = *O*-aminohexylated d-tyrosine; FITC = fluorescein

	Premise	CPP	Inhibiting factor	Cargo	Trigger	Ref.
pH sensitive conformational changes						
1	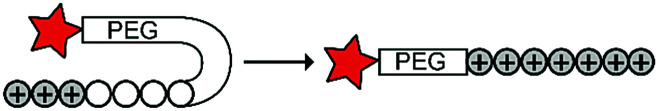	Tat	PEG	DOX loaded micelle	pH drop	63
2	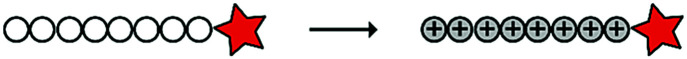	TH	H side chain charge	CPT	pH drop	64
		LH		CPT		66
		TH		PTX probe		65
3	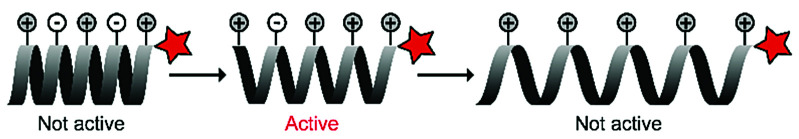	K_97_	SC modi-fications with imi-dazole or carboxylic acids		pH drop to 6.0	67

Light sensitive conformational changes						
4	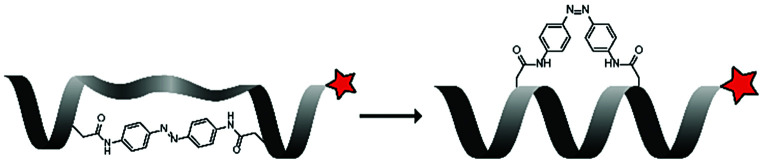	LK	*trans*-Ab	Tamra	365 nm, 5 min, 8 mW cm^−2^	68
5	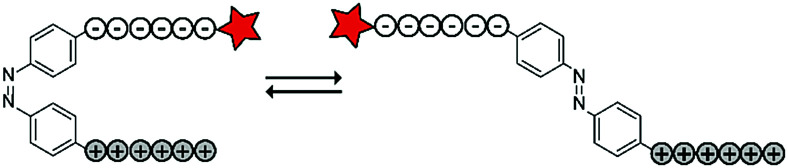	R_9_	E_9_	RhoB	*λ* = 488, 1–1.5 μW, ∼60 ms μm^−2^	69
6	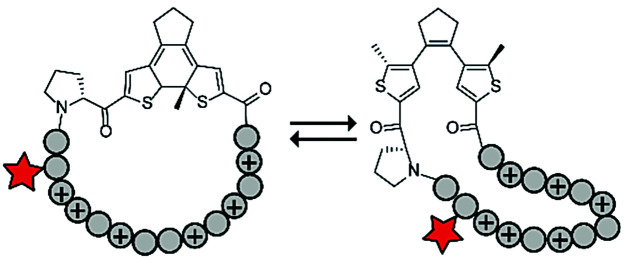	RRF-y^Ahx^P-FRR	Circular structure	FITC	*λ* = 590 nm	70

Both the Wang group and He group developed ACPPs by exchanging all lysine residues in two lysine-rich CPPs for histidines, creating respectively TH (AGYLLGHINLHHLAHLXHHIL, with X = 2-aminoisobutyric acid)^64,65^ and LH (LHHLLHHLHHLLHH, Table 4, entry 2).^66^ At physiological pH, neither peptide is able to enter cells, unlike their lysine rich counterparts (TK and LK, respectively). Acidification to pH 6 protonated the histidine residues and induced cellular uptake. With this system, the Wang group delivered anticancer drug camptothecin intracellularly with both peptides – TH for HeLa cells and LH for human breast adenocarcinoma cells.^64,66^ The He group coated TH onto liposomes loaded with either the drug paclitaxel or IR-probes.^65^ Paclitaxel was delivered to tumorigenic tissue *in vitro*, as demonstrated by flow cytometry and cellular imaging, while NIR imaging showed *in vivo* delivery of IR-probes to the tumour sites.

The Kim group harnessed α-helical conformational requirements in their ACPP design. They introduced imidazole and carboxylic acid groups onto lysine residues to ensure pH-dependent conformational control of a synthetic, polylysine-based CPP (Table 4, entry 3).^67^ At physiological pH, the charge distribution created an inactive, tightened helix, while in a slightly acidic environment (pH 6) the partial protonation gave rise to an intact helical structure that could enter cells. When the pH dropped below 4, however, additional protonation stretched the helix further through strong intramolecular electrostatic repulsion that led to inhibition of uptake. They demonstrated the potential of the approach by showing that the ACPPs were taken up in human lung carcinoma cells at pH 6, but not at pH 7.4.

### Light triggered conformational changes

Under ambient conditions, the azobenzene (Ab) photoswitch exists in a low-energy *trans*-conformation that can isomerize to a *cis*-conformation upon irradiation with UV light. The group of Lee used this to control the helicity of a leucine and lysine rich peptide (LK), by inserting an azobenzene bridge between the side chains of two cysteine residues in the peptide sequence (LKKLLKC̲LKKC̲LKLAG, Table 4, entry 4).^68^ The *trans*-azobenzene stretches the CPP and disrupts its biologically active structure. UV-irradiation (365 nm) generates the *cis*-conformation which pulls the CPP into an α-helical structure that is suitable for uptake. FACS showed that irradiated – mostly *cis*-Ab – peptide was taken up in 76% of HeLa cells, while nonirradiated – mostly *trans*-Ab – peptide was taken up in only 27% of the cells. This uptake behaviour was corroborated with confocal microscopy when irradiation was applied *in situ* for 5 minutes.

The Möller group placed the azobenzene linker in between an oligo-arginine and an oligo-glutamate domain to enable the reversible activation of the CPP using light. The authors included a fluorescent rhodamine B (RhoB) label for visualization and evaluation of the uptake of the construct (RhoB-E_9_-Ab-R_9_, Table 4, entry 5).^69^ Irradiation of the azobenzene with UV light to the *cis*-conformation (*λ* = 366 nm, 20 min) resulted in a parallel configuration of the two peptide chains and inactivation of the CPP. Repeated irradiation with longer wavelength laser light (*λ* = 488, 1–1.5 μW, ∼60 ms μm^−2^) recovered a *trans*-conformation and restoring CPP activity. With this construct, high spatial control could be achieved as cellular uptake was only observed in 488 nm irradiated areas without inducing cellular toxicity as evidenced by confocal microscopy and quantified by flow cytometry.

Photoswitches have also been combined with benign red light activation. The Ulrich group designed circular ACPPs consisting of a peptide fragment of 9 to 14 residues, with a charge between +4 and +10, and a photoswitchable diarylethene (DAE) group (Table 4, entry 6).^70^ DAE is a rigid structure that becomes flexible upon irradiation with visible light (*λ* = 590 nm), while irradiation with UV-light brings the DAE to its rigid form. The rigid DAE imposed a non-optimal structure for the circular ACPP thereby preventing it from entering the cell. Irradiation with visible light induced a conformational change that enabled cellular penetration, which increased 1.6 to 6.5 fold depending on the peptide sequence.

## CPP activation through *in situ* conjugation

Besides modifying the structure or conformation of the parent peptide to control uptake properties, more recent approaches focussed on merging inactive peptide fragments to construct an active CPP. This approach allows targeting of individual inactive peptide fragments, *e.g.* by using a targeting antibody or molecule, before constructing and activating the CPPs for cellular uptake at the site of interest. The strategies to create this type of ACPPs are described below and summarized in Table 5.

### Fusion of two peptide fragments

In an early report, the Löwik group explored the split CPP strategy by merging two peptide fragments into a functional CPP using *in situ* disulfide bridge formation (Table 5, entry 1).^71^ Truncated peptide fragments with a length of three (R_3_), four (R_4_), or five (R_5_) arginine residues were synthesized and terminated with a cysteine residue to allow disulfide bridge formation and a FITC moiety for visualisation and quantitative analysis. The length of the resulting full size CPPs ranged from eight to ten arginine residues. Although they showed that the disulfide bridge was stable in the extracellular environment, it is likely reduced in an intracellular environment. All ACPPs showed uptake in HeLa cells, while the symmetric structures (R_4_–R_4_ or R_5_–R_5_) gave the best results. Although cellular uptake studies *in vitro* were successful, free extracellular thiols might interact with reactive groups *in vivo* when used in combination with targeting moieties and thereby trapping and inactivating the individual CPP halves.

As an alternative conjugation strategy, the Löwik group explored the construction of active CPPs using leucine zippers.^72^ Leucine zippers are α-helical structural motifs found in proteins that dimerise when in close contact.^73^ For the design of these heterodimeric zipper-based ACPPs, monomeric zippers were conjugated to either a tetraarginine or a fluorescently labelled tetraarginine. Assembly of the peptide zippers resulted in the formation of a semi-linear octaarginine chain and subsequent uptake in HeLa cells (Table 5, entry 2). The authors further showed that using these zipper constructs, not only low molecular weight FITC but also superfolder green fluorescent protein (27 kDa) could be transported into the cells.

In a follow up study, the authors assembled ACPPs using bioorthogonal chemistry. This chemistry is widely used for a variety of bioconjugation applications *in vitro* and recent developments of the bioorthogonal reactants has also made this chemistry applicable *in vivo*.^74^ In this case, tetraarginines were conjugated to various bioorthogonal handles that could be used for an *in situ* conjugation *via* the inverse electron-demand Diels–Alder reaction with tetrazines, one of the fastest bioconjugation reactions known to date (Table 5, entry 3).^75^ The authors modified one tetraarginine half with a fluorophore and a tetrazine (Tz) and another half with a bicyclo[6.1.0]nonyne (BCN) or a *trans*-cyclooctene (TCO) moiety. Upon 30 minutes of incubation of the two halves, a level of cellular uptake was observed comparable to that of pre-conjugated CPP halves or to the native octaarginine CPP as evidenced by confocal scanning laser microscopy and flow cytometry. Moreover, using a slightly longer incubation time (90 minutes) and a moderately increased peptide concentration (10 μM versus 5 μM), the BCN–Tz delivery system was successfully used to deliver the 66 kDa human serum albumin protein into HeLa cells.

### Conjugation through polymerization

In an alternative approach, the group of Matile reported on-site ring-opening disulphide-exchange polymerization to obtain cell-penetrating poly(disulphides) (CPDs) (Table 5, entry 4).^76,77^ Upon cell entry, the disulphide backbone dissociates to the individual monomers by the reducing intracellular environment. CPDs with fluorescent cargo were taken up into HeLa cells *in vitro*, as visualized by confocal microscopy. Furthermore, DOX,^78^ various proteins,^78,79^ antibodies,^78,79^ quantum dots^80^ and mesoporous silica nanoparticles^81^ were also successfully delivered *via* this strategy. However, *in vivo* applicability and target specificity have not been assessed yet.

The Gianneschi group described activation of cellular uptake through polymerization of peptides into high density bushes.^82^ They attached either one or two arginine or lysine residues to a short peptide sequence without any positively charged amino acids (GSGSG) and that lacks cell penetrating properties. The peptides were equipped with norendimide moieties, and the resulting alkene monomers were polymerized with oligoethelyne glycol to form block copolymers. Peptide oligomers with a degree of polymerization (DP) of 60 – resulting in 60 or 120 positive charges depending on addition of one or two positive amino acids – showed cellular uptake in HeLa cells (Table 5, entry 5). This strategy was expanded to the non-internalizing, lysine rich, apoptotic peptide KLAK. When this peptide was conjugated to the polymer (DP = 5, 10, 15), again, cellular uptake was observed through flow cytometry while the apoptotic properties of KLAK remained intact in a dose-dependent manner. It should be noted, however, that the structures were polymerized before administration and could not be activated on site. To evolve this strategy to its full potential with respect to ACPP design, the possibility of targeted on site polymerization is essential. Such a strategy may provide a potent approach for transportation of therapeutic peptides across the cell membrane.

**Table 5:** Overview of activatable cell-penetrating peptides (ACPPs) triggered through conjugation. Amino acids are indicated *via* the single letter code, in which d-amino acids are noted in lower case. FITC = fluorescein; GFP = green fluorescent protein; HSA = human serum albumin; QD = quantum dots; BRD-4 = bromodomain-containing protein 4; CASP-3 = caspase 3; BSA = bovine serum albumin; Ab = Alexa Fluor 488 goat anti-rabbit IgG; MSN = mesoporous silica nanoparticle; DP = degree of polymerization

	Premise	CPP	Cargo	Ref.
Fusion of two subunits				
1	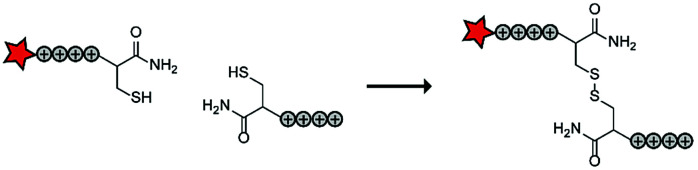	R_8_	FITC	71
2	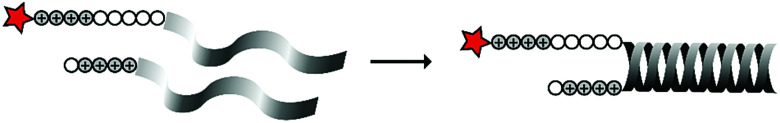	R_8_	FITC GFP	72
3	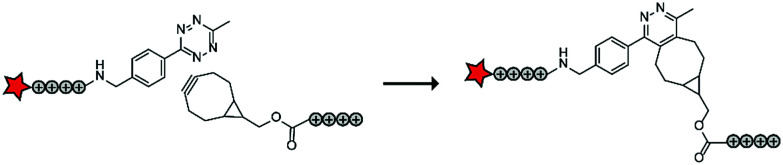	R_8_	FITC HSA	75

Polymerization				
4	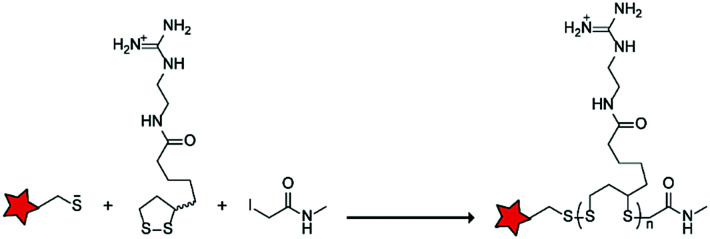	Poly Arg side chains	FITC or Tamra	76
			FITC	83
			DOX, avidin, BRD-4, CASP-3, BSA, Ab	78
			BSA, IgG	79
			QD	80
			MSNs	81
5	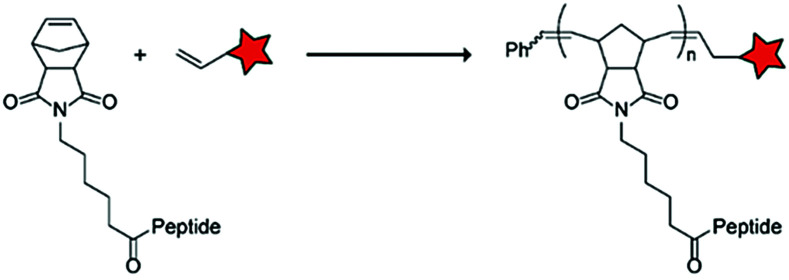	GSGSG	KLAK	82
		KLAK		
		DP > 5		

## Summary and outlook

The increasing number of reports describing the different approaches to control CPP activity is promising for their future use as drug delivery vehicles. Altogether, the current strategies cover a wide array of applications.

CPPs can be temporarily inactivated by introducing interacting inhibitory domains or, when bulky groups are undesired, smaller side chain modifications on, for example, lysine residues to mask CPP activity. Masking groups can be removed by local triggers such as enzymes or changes in pH as well as external triggers such as light. While several approaches are summarized in this review, the list of sensitive linkers and possible triggers is far from exhausted. Besides unidirectional activation through removal of inhibitory moieties, reversible activation has also been achieved by controlling the peptide conformation.

Unfortunately, the triggers used to activate CPPs are not that binary *in vivo*. Enzymes that are overexpressed in diseased tissue may still be present in lower amounts in healthy tissue, and gradient pH values are often observed between tissues. CPP activation by irradiation with light is beneficial to create temporal and special control, but is challenged by the poor tissue penetration depth as well as the potential cellular toxicity induced by the harmful wavelengths. These unintended triggers may create off-target effects and should be accounted for. Nevertheless, some of the research covered in this review demonstrated the benefit of using ACPPs *in vivo*.

Activation of CPPs by conjugating two inactive peptide halves does not require an environmental trigger. Spatial control of CPP activity can be achieved by including a targeting entity on one of the peptide halves, such as an antibody or localizing small molecule. Here, the targeted fragments localize to the target site of interest, after which their counterparts interact and prime them for cellular uptake. Using bioorthogonal chemistry, such a pre-targeting approach has already been used to increase, for example, the radiolabelling specificity *in vivo*.^84^ Successful *in vivo* conjugations rely on the availability of highly stable reactants and reactions with exceptional high rate constants as the reactant concentration *in vivo* are low.

CPPs have entered clinical trials for treatments of several dysfunctions including hearing loss,^85^ coronary artery disease,^86^ macular degeneration,^87^ solid tumours,^88^ central nervous tumours,^89^ scar prevention,^90^ heart attack,^91^ Duchenne muscular dystrophy,^92^ and ocular inflammation.^93^ However, to date, none have been approved for therapeutic use. CPP application in the clinic is challenged by the limited bio-distribution and accumulation of the structures in liver or kidney.^17^ In addition, the half-life of the structures varied from 1.2 to >72 hours, where short lived CPPs generally contained cationic residues likely making them more susceptible for proteolytic cleavage. Adopting ACPP strategies as described in here may improve the stability and bio-distribution of the constructs and therefor also possible clinical translation.

To the best of our knowledge, one ACPP has been tested in a phase 1 clinical study and used for imaging purposes.^94^ This ACPP was designed to visualize tumours during surgical procedures and resembled the ACPP designed by the Tsien group, containing an inhibitory domain and a protease-sensitive linker.^19,95,96^ In this construct, the CPP as well as the inhibitory domain each carried a fluorophore thereby inducing FRET. Proteolytic cleavage in tumorigenic tissue disabled FRET resulting in a measurable change of fluorescence intensity. The ACPP was administered via intravenous infusion for 30 minutes, 2 to 20 h before the surgery and allowed the discrimination between tumour-positive and tumour-negative tissue with limited adverse events. This positive application and the increased specificity of ACPPs over native, non-activatable CPPs greatly improves their possible use for other applications *in vivo*. Keeping in mind the variety of ACPPs and their activation triggers, we foresee promising clinical potential for the local cellular delivery of a variety of therapeutics using these structures.

## Conflicts of interest

There are no conflicts to declare.

## Supplementary Material
